# Impaired Peripheral Blood Mononuclear Cell (PBMC) Mitochondrial Respiration Is Associated with Mortality and Long COVID Syndrome Severity in COVID-19 Patients

**DOI:** 10.3390/ijms262110377

**Published:** 2025-10-24

**Authors:** Anne-Laure Charles, Léa Debrut, Walid Oulehri, Véronique Vincent, Héloise Delagreverie, Pauline Asael, Marianne Riou, Margherita Giannini, Alain Meyer, Bernard Geny

**Affiliations:** 1 Biomedicine Research Center of Strasbourg (CRBS), UR 3072, “Mitochondria, Oxidative Stress and Muscle Plasticity”, Faculty of Medicine, University of Strasbourg, 67081 Strasbourg, France; anne.laure.charles@unistra.fr (A.-L.C.); ldebrut@unistra.fr (L.D.); walid.oulehri@chru-strasbourg.fr (W.O.); pasael@unistra.fr (P.A.); marianne.riou@chru-strasbourg.fr (M.R.); margherita.giannini@chru-strasbourg.fr (M.G.); alain.meyer1@chru-strasbourg.fr (A.M.); 2 Department of Anesthesia and Intensive Care, Strasbourg University Hospital, 67091 Strasbourg, France; 3 Department of Physiology and Functional Exploration, Strasbourg University Hospital, 67091 Strasbourg, France; veronique.vincent@chru-strasbourg.fr; 4 Department of Clinical Microbiology, Hôpital Avicenne AP-HP, 125 rue de Stalingrad, 93000 Bobigny, France; 5 Faculté de Médecine, INSERM U1137 IAME, Université Sorbonne Paris Nord and Université Paris Cité, 16 rue Henri Huchard, 75018 Paris, France

**Keywords:** COVID, long COVID, peripheral blood mononuclear cells (PBMC), mitochondria, mitochondrial respiration, inflammation

## Abstract

COVID-19 is a public health issue with a significant mortality rate and potential long-lasting disabling symptoms responsible for the long-COVID syndrome. Mitochondrial dysfunction is a key mechanism but whether peripheral blood mononuclear cell (PBMC) mitochondrial respiration changes might be associated with mortality and/or occurrence and severity of long-COVID syndrome remains to be investigated. We determined mitochondrial respiratory chain oxygen consumption in twenty COVID-19 patients hospitalized in the intensive care unit and analyzed their remaining symptoms at the third year after hospital release. PBMC mitochondrial respiration was decreased in COVID-19 patients compared to the control group (14.13 ± 2.35 vs. 6.21 ± 0.88 pmol/s/10^6^ cell, *p* = 0.0006 for the OXPHOS state by CII). Considering COVID severity, such a decrease was greater in long-COVID patients and in patients who deceased (4.91 ± 0.75, *p* = 0.008 and 4.94 ± 1.11 pmol/s/10^6^ cell, *p* = 0.04, respectively). PBMC markers of inflammation also increased with the severity of COVID (1.0 ± 0.08 vs. 14.45 ± 2.07, *p* = 0.02 for ISG15 in patients who died) and ISG15 negatively correlated with PBMC mitochondrial respiration (r = −0.67, *p* = 0.02 for CII). In conclusion, this study shows that the greater the impairment in PBMC mitochondrial respiration in patients hospitalized in the intensive care unit for COVID-19, the greater the mortality rate and the more severe the long-COVID symptoms—three years after hospital discharge. Further, PBMC markers of inflammation also increased with the severity of COVID and ISG15 negatively correlated with PBMC mitochondrial respiration. These results support that PBMC mitochondrial respiration might be a biomarker of COVID severity and further studies investigating whether modulation of PBMC mitochondrial respiration might improve COVID-19 patients’ prognosis.

## 1. Introduction

The SARS-CoV-2 virus was discovered in November 2019 and is responsible for the worldwide COVID-19 pandemic. Until today, this virus infected more than 760 million people, resulting in at least 6.9 million deaths. This is one of the worst pandemics that humanity suffered from. Apart from acute effects, a significant proportion of people recovering from acute SARS-CoV-2 infection present with long-COVID syndrome. Thus, as many as 50% of the patients can present long-hauling symptoms several months after infection and around 10% of the patients contaminated with SARS-CoV-2 still present with prolonged symptoms even years later [1,2,3,4,5,6]. The patients’ symptoms are numerous and cannot be explained by other diagnosis. Moreover, symptomatology can fluctuate over time, potentially leading to altered quality of life and to persistent unemployment [1,2,3,4,5,6,7,8,9,10,11,12]. The symptoms of long-COVID occur across multiple organ systems and among others, include fatigue, cognitive impairment, cough, dyspnea, diarrhea, dysgeusia, dysosmia, fever, exercise intolerance, headaches, and pain [9,10,11,12,13,14,15,16], suggesting a pathophysiology of multifactorial origin.

The cellular aspects of COVID are not entirely understood. Damaged tissues and organs are related to inflammation, thrombosis, and/or autoreactive B and T cells, together with vascular dysfunction resulting from—or aggravated by the SARS-CoV-2 virus [13,17,18]. More precisely, immune dysregulation associated with mitochondrial dysfunction is thought to be implicated in COVID-19 deleterious effects. Thus, mitochondrial dysfunction has been described in acute severe SARS-CoV2 infection, including altered bioenergetics, reduced oxidative phosphorylation, altered mitochondrial ultrastructure and decreased proton leak. Further, mitochondrial dysfunctions in the lung, heart and skeletal muscles were associated with symptoms related to cardiorespiratory insufficiency and muscle impairments [5,19,20,21,22,23].

The study of peripheral blood mononuclear cells (PBMC) is particularly interesting since PBMC participates in immune and inflammatory responses, key pathogenic mechanisms in COVID infection. During acute COVID, a reduced oxygen consumption rate, mitochondrial depolarization, shift to glycolysis and abnormal mitochondrial morphology have been observed in PBMC [20,21,24]. Nevertheless, the potential involvement of the mitochondrial respiratory chain complex respiration in PBMC in the prognosis of patients suffering from COVID deserves further study, not only considering mortality rates but also considering long-COVID occurrence and severity.

The aim of this study was therefore to determine whether peripheral blood mononuclear cells’ mitochondrial respiration changes might be associated with mortality and/or with the occurrence and severity of long-COVID syndrome. For this purpose, we investigated the PBMC mitochondrial respiratory chain complex respiration, together with markers of inflammation, in COVID-19 patients hospitalized in the intensive care unit. We also investigated a potential relationship between PBMC characteristics and patients’ mortality rate on the one hand, with occurrence and severity of long-COVID the third year after hospital release.

## 2. Results

### 2.1. Clinical and Biological Characteristics of the Population

Table 1 summarizes the demographics and clinical characteristics of the population, composed of twenty COVID-19 patients and eight healthy volunteers. The 20 patients with confirmed COVID-19 diagnosis were admitted in the intensive care unit at the Hospital of Strasbourg (France). The mean age was not significantly different between these two groups (59.49 ± 1.10, vs. 65.69 ± 1.69 years). The body mass index (BMI) of the COVID-19 patients was significantly higher compared to that of the healthy volunteers (25.00 ± 1.60, vs. 29.50 ± 1.33 kg/m^2^, *p* = 0.03). As expected, the healthy subjects showed no risk factors, the most frequent being systemic hypertension, diabetes and dyslipidaemia in the COVID patients.

To go further, we classified the COVID patients depending on the severity of the disease, ranging from long-COVID with few (n = 6) or many symptoms (n = 6), to mortality (deceased patients, n = 4).

The main biological characteristics of the patients are presented in Table 2, as compared to the normal laboratory values. As compared to controls, COVID patients presented mainly with anemia and enhanced inflammatory markers, including increased C-reactive protein (CRP) and neutrophil-to-lymphocyte ratio (NLR) and decreased leucocyte-to-lymphocyte ratio (LLR).

### 2.2. Long-COVID Symptoms Characterization

Three years after hospital discharge, we conducted phone calls to determine eventual symptoms corresponding to long COVID. We asked the patients to rank their symptoms on a scale ranging from 0 to 10 (0 means no symptoms and 10 means the highest intensity of symptoms). Out of the 20 initial patients, four had died and four did not respond. In the 12 responding patients, the total score was calculated by summing each individual symptom score. We classified the patients based on COVID severity. Patients with a total score of post-COVID symptoms <10 were classified as no or few post-COVID symptoms. Two patients presented no remaining symptoms three years after hospital release. Patients with a total score of post-COVID symptoms >10 were classified as many post-COVID symptoms.

Table 3 shows that anosmia/agueusia, dyspnea, tiredness and muscle pain were the most common and the strongest symptoms in the patients presenting with long COVID.

**Figure 1:**
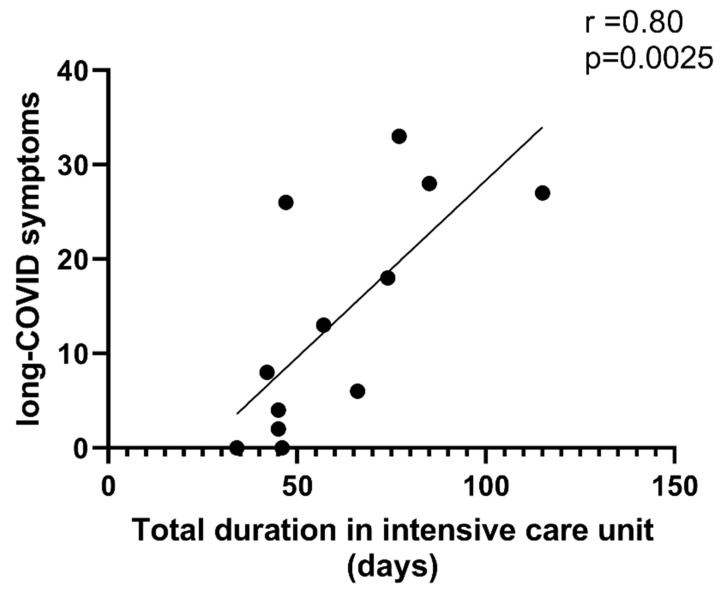
Severity of long-COVID symptoms positively correlated with the length of stay in the intensive care unit (n = 12).

Interestingly, we observed a positive correlation between the length of stay in the intensive care unit and the post-COVID severity (*p* = 0.0025, Figure 1).

### 2.3. PBMC Mitochondrial Respiration

**Figure 2:**
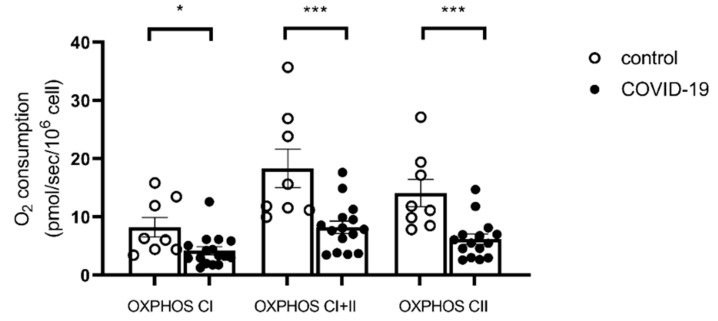
Mitochondrial respiration is impaired in patients with COVID-19 compared to controls. Mitochondrial respiratory capacity of oxidative phosphorylation (OXPHOS) by CI, OXPHOS by CI&CII, and OXPHOS by CII on isolated PBMCs. All values are expressed as the mean ± SEM (n = 8 for CTL group, n = 16 for COVID-19 group. * *p* < 0.05; *** *p* < 0.001. See figure in methods for a detailed explanation on substrate use.

Considering the entire population, the OXPHOS state by CI was decreased in the COVID-19 group compared to the control group (8.24 ± 1.68 vs. 4.03 ± 0.62 pmol/s/10^6^ cell, *p* = 0.007, Figure 2). A similar impairment was observed in the COVID-19 group compared to the control group during OXPHOS CI + II respiration (18.34 ± 3.33 vs. 8.24 ± 1.06 pmol/s/10^6^ cell, *p* = 0.0008). The OXPHOS CII state also decreased in the COVID-19 group compared to the control group (14.13 ± 2.35 vs. 6.21 ± 0.88 pmol/s/10^6^ cell, *p* = 0.0006).

To investigate in more detail a potential relationship between COVID severity and PBMC mitochondrial respiration, we classified the patients in three subgroups, depending on COVID severity (patients with few or many post-COVID symptoms and deceased patients).

**Figure 3:**
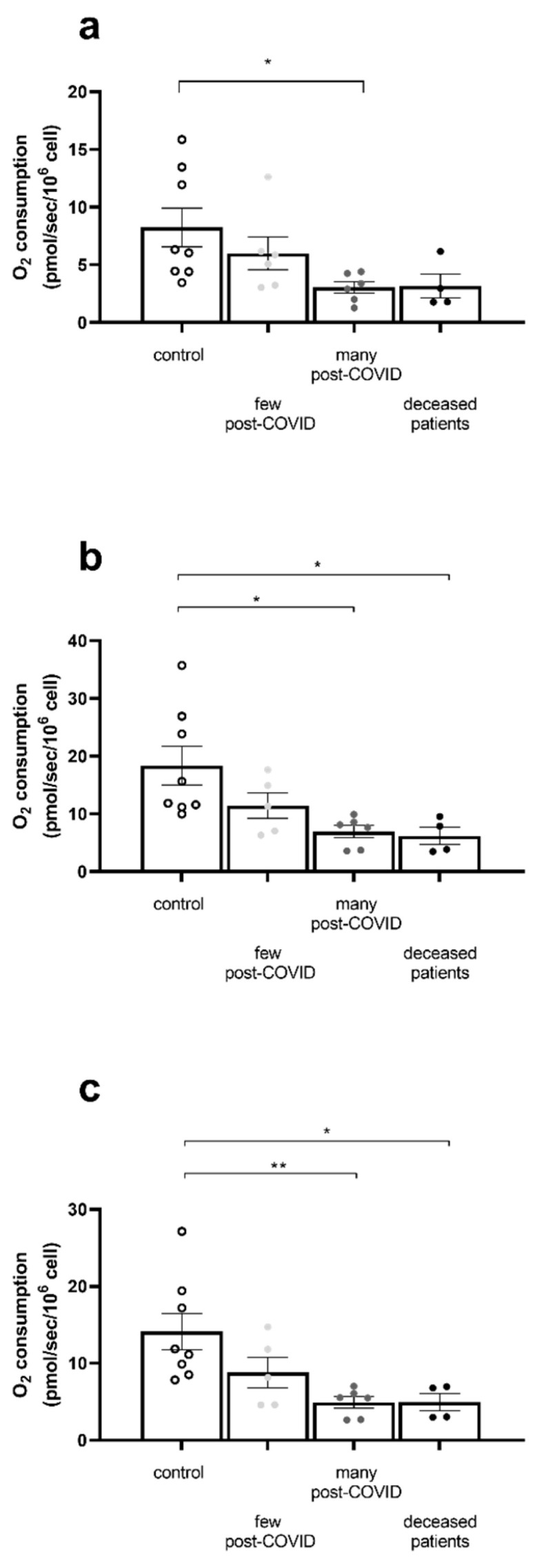
PBMC mitochondrial respiration impairment is greater in patients that deceased or present with severe long-COVID symptoms. (**a**) Oxidative phosphorylation (OXPHOS) by CI, (**b**) OXPHOS by CI + CII, and (**c**) OXPHOS by CII on isolated PBMCs. All values are expressed as the mean ± SEM (n = 8 for CTL group, n = 6 for few post-COVID and n = 6 for many post-COVID). * *p* < 0.05; ** *p* < 0.01. See figure in methods for a detailed explanation on substrate use.

OXPHOS CI was significantly reduced in the COVID group with high post-COVID symptoms, compared to the control group (8.24 ± 1.69 vs. 3.04 ± 0.51 pmol/s/10^6^ cell, *p* = 0.037, Figure 3a).

We observed the same profile for the OXPHOS CI + II state. A significant mitochondrial respiration decrease was found in the patients with many post-COVID symptoms group and in patients who died, compared with the control group (6.93 ± 1.08 and 6.19 ± 1.5 vs. 18.34 ± 3.33 pmol/s/10^6^ cell in the control group, *p* < 0.05 for each comparison with the control group, Figure 3b).

The OXPHOS state by CII decrease was also significant in the many post-COVID symptoms group compared to the control group (14.13 ± 2.35 vs. 4.91 ± 0.75, *p* = 0.008). Such a significant impairment was also observed in the deceased patients (4.94 ± 1.11, *p* = 0.04, Figure 3c).

### 2.4. Inflammatory Marker Transcripts Are Enhanced in the Most Severe COVID Patients

**Figure 4:**
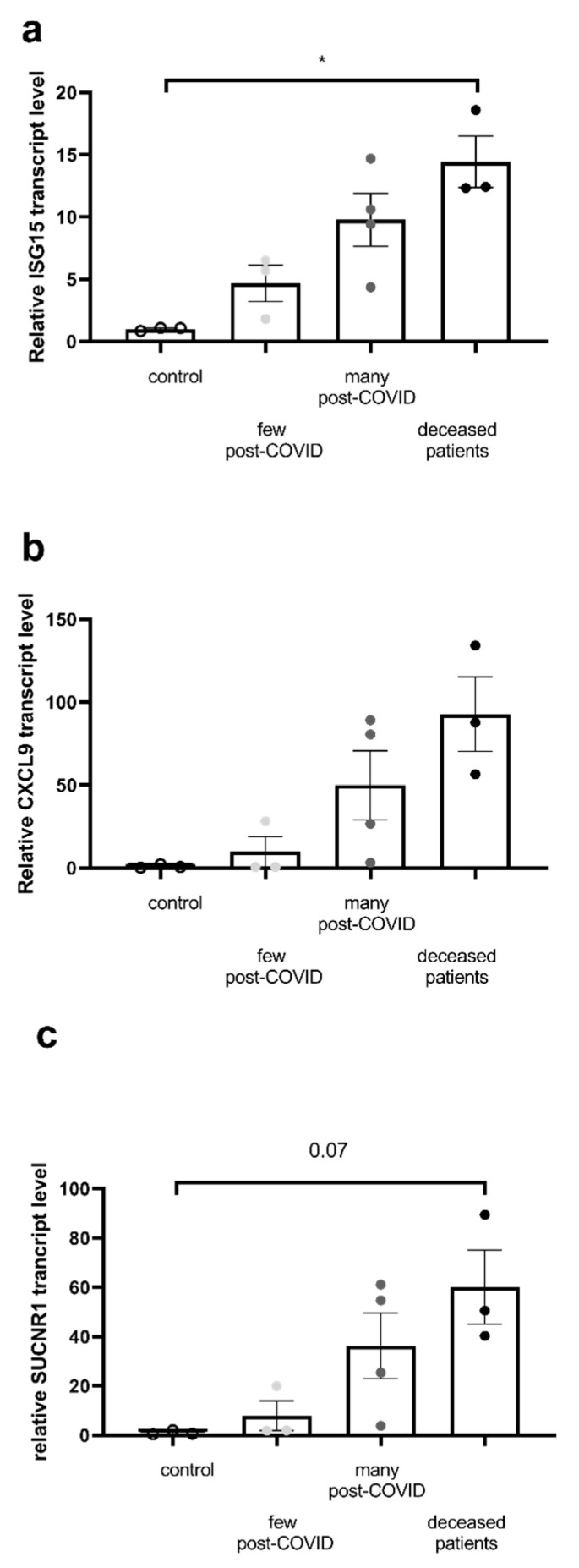
Inflammatory markers are increased in the most severe COVID patients. Relative transcript levels of (**a**) ISG15, (**b**) CXCL9, (**c**) SUCNR1. Results are expressed as mean ± SEM. The levels were normalized to the YWHAZ gene (housekeeping). ISG15: interferon-stimulated gene 15; CXCL9: C-X-C motif chemokine ligand 9, major role in the adaptive immune response against infections and tumors. SUCNR1: succinate receptor 1, G protein-coupled receptor activated by succinate. All values are expressed as the mean ± SEM (n = 3 for CTL group, n = 3 for few post-COVID, n = 4 for many post-COVID, and n = 3 for deceased patients). * *p* < 0.05.

To investigate the potential mechanisms involved in the greater PBMC mitochondrial dysfunction, we determined whether inflammatory and oxidative stress markers are enhanced in the more severe COVID patients. Since enhanced inflammatory pathways are known to participate in COVID severity, we examined in PBMC the expression of important genes involved in inflammation such as interferon-stimulated gene 15 (ISG15), chemokine ligand-9 (CXCL9), and the gene expression of the succinate receptor 1, SUCNR1 (Figure 4).

All markers tended to be increased in relation to COVID severity, the increase in ISG15 transcript being significant in patients who died (1.0 ± 0.08 vs. 14.45 ± 2.07, *p* = 0.02).

**Figure 5:**
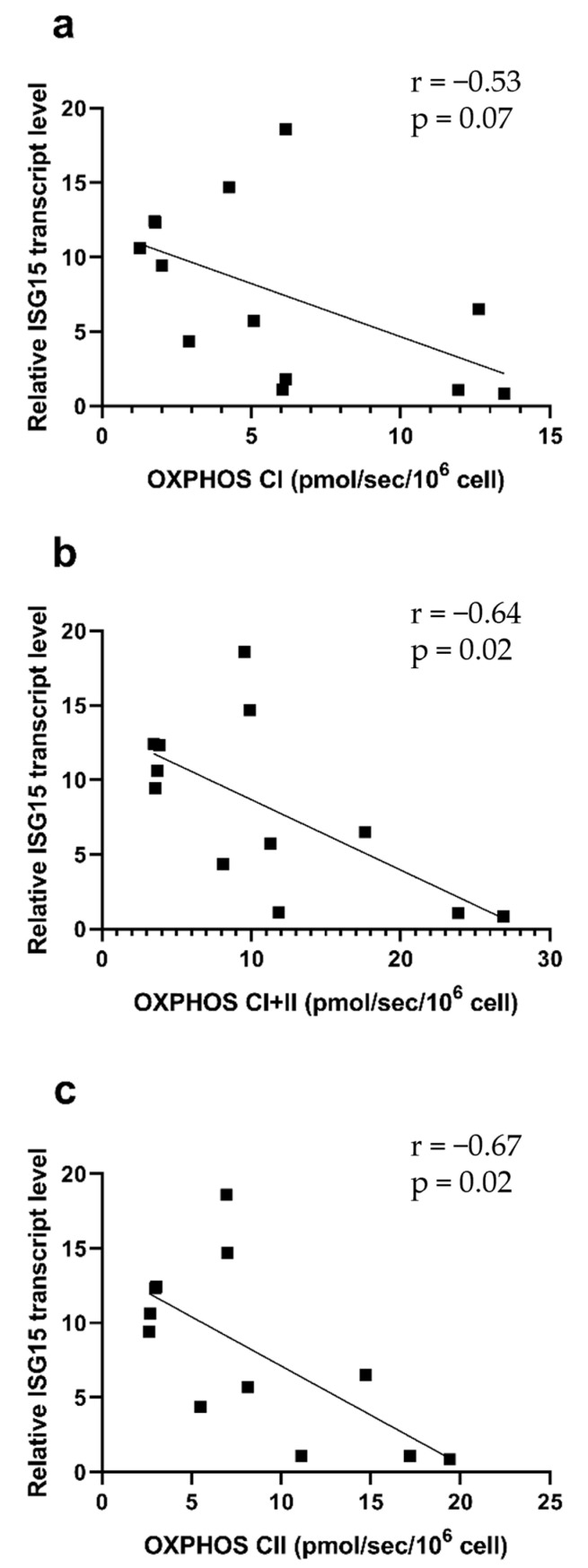
Correlation between mitochondrial respiration and the relative ISG15 transcript level. (**a**) OXPHOS by CI, (**b**) OXPHOS by CI and CII, (**c**) OXPHOS by CII on isolated PBMCs. Results are expressed as mean ± SEM (n = 12).

Finally, we observed significant negative correlations between the ISG15 transcript and PBMC mitochondrial respiration (Figure 5).

## 3. Discussion

The main result of this study is the demonstration that the greater the impairment in PBMC mitochondrial respiration, the greater the mortality rate and the more severe the long-COVID symptoms in patients hospitalized in the intensive care unit for COVID-19—three years after hospital discharge.

### 3.1. PBMC Mitochondrial Respiration Is Impaired in COVID-19 Patients

In our COVID-19 patients, we observed impaired PBMC mitochondrial respiration, involving several complexes of the mitochondrial respiratory chain. This is consistent with previous data in circulating cells [19]. Indeed, mitochondrial dysfunction has been observed in acute SARS-CoV-2 infection [20]. Ajaz et al. reported that COVID patients were characterized by mitochondrial dysfunction with energy deficit, leading to a metabolic switch toward glycolysis [21]. Similarly, De Vitis et al. also observed decreased PBMC mitochondrial respiration in COVID patients, as compared to healthy controls [25]. Particularly, the authors suggested that the mitochondrial respiration impairment, associated with reduced FT3 serum levels, may have hampered their reaction to viral infection [25]. Interestingly, although the degree of mitochondrial dysfunction likely depends on the COVID-19 stage and associated organ damages [22,26], this might have clinical significance since the virus reduces mitochondria-related antiviral-signaling pathways [19,20,21,22,27,28,29,30]. Although all mechanisms involved in severe and/or long-COVID symptoms still need investigation, mitochondrial complications are likely related to spike protein. Thus, SARS-CoV-2 spike protein decreases energy production, disrupts mitochondrial dynamics and inhibits recycling of the mitochondria by mitophagy through a possible downregulation of TOM20 [31,32,33,34]. Nevertheless, whether such mitochondrial dysfunction is associated with increased mortality and/or occurrence and severity of long COVID still deserves further investigation.

### 3.2. Mortality Is Associated with Severe Impairment in PBMC Mitochondrial Respiration

The finding of prognostic factors is important to adapt medical therapy aiming to reduce disease-induced mortality. Besides clinical risk factors, such as clinical severity and the related hospital length of stay, several parameters were linked with increased mortality in COVID-19 patients. Thus, hospital-based mortality was associated with solid organ tumors, diabetes, renal, liver and cardiorespiratory diseases [35].

Increased inflammatory markers and/or factors related to endothelial and other organ dysfunctions were also associated with a worse prognosis [36,37,38]. Accordingly, increased NLR was shown to be associated with a greater risk of intra-hospital mortality [36,39].

In our study, inflammatory markers were augmented in the most severe COVID patients, and particularly ISG15 was significantly increased in COVID patients who eventually died. This is in line with previous data showing an enhanced expression of ISG15 in the lungs and blood of severe COVID patients [40]. Similarly, CXCL9, a chimiokine associated with gamma interferon and SUCNR expression, stimulated during hypoxia [41], tended to be increased as compared to controls and to patients with no or few post-COVID symptoms.

Concerning PBMC, peripheral blood monocytes from patients with COVID-19 pneumonia demonstrated altered bioenergetics and reduced basal, maximal and spare mitochondrial respiratory capacity [20]. Fibroblast growth factor 21, proposed as a biomarker of mitochondrial dysfunction, was also shown to be associated with COVID-19 severity and mortality [21]. We therefore challenged the hypothesis that impaired PBMC mitochondrial respiration might be associated with COVID-19 patients’ mortality. Indeed, the patients who died had a lower level of PBMC mitochondrial respiration. This result further supports the involvement of the PBMC metabolism in COVID pathophysiology.

### 3.3. Severe Long-COVID Symptoms Are Associated with Severe Impairment in PBMC Mitochondrial Respiration

Interestingly, long COVID was associated with lactic acidosis and chronic muscle weakness, suggesting alterations in mitochondrial oxidative phosphorylation. De Boer et al. observed decreased fat oxidation and increased lactate production in a cohort of long-COVID patients [37,38]. Such abnormalities are likely to be involved in patients’ tiredness and muscle damages associated or not with sarcopenia [42].

Concerning PBMC, several pieces of data supported modified mitochondrial respiration in long-COVID patients [38,43], but whether impaired PBMC mitochondrial respiration might be a biomarker of later occurrence and severity of long COVID was unknown.

Although a causal relationship remains to be proven, our data supports a link between PBMC mitochondrial respiration and long-COVID occurrence and severity. Indeed, the patients demonstrating the greater number and severity of symptoms were the patients with the lower PBMC mitochondrial respiration. This was true for both OXPHOS I and II-related mitochondrial respiration. To the best of our knowledge, such findings between impaired PBMC mitochondrial respiration and subsequent occurrence and severity of long COVID have never been reported.

To investigate further potential molecular mechanisms, we determined whether PBMC inflammatory and interferon pathways might be related to mitochondrial respiration. Although not reaching statistical significance, such parameters tended clearly to be increased in patients with many post-COVID symptoms (i.e., long-COVID patients). Further, ISG15 negatively correlated with PBMC mitochondrial respiration.

Supporting our results, Marzetti et al. recently reported an improvement in mitochondrial quality in long-COVID patients supplemented with beetroot juice, associated with increased physical performance and endothelial function [44].

### 3.4. Limitations of the Study

First, the BMI and age of the controls were slightly higher than that of COVID patients, which may be considered a limitation of the study. Second, several patients suffered from chronic respiratory failure, associated with more symptoms. Although chronic situations are different from acute situations, this characteristic could have modulated our results. Indeed, we previously reported that PBMC mitochondrial respiration was enhanced in severe asthma patients in exacerbation [44]. In contrast, PBMC respiration was decreased in patients with chronic obstructive pulmonary disease [45,46]. Nevertheless, the small number of patients in this subgroup prevents further analysis and justifies a larger-scale study to particularly examine the participation of respiratory failure in PBMC mitochondrial dysfunction.

## 4. Methods

### 4.1. Population and Study Design

Twenty patients, hospitalized in the intensive care unit for COVID-19, participated in this prospective study. COVID-19 was diagnosed by positive real-time reverse-transcriptase polymerase chain reaction from a nasal and/or throat swab together with clinical signs, symptoms, and/or radiological findings suggestive of COVID-19 pneumonia. Data regarding COVID-19-related hospitalization were collected by systematic medical chart review, and long-COVID symptoms were obtained by performing a phone call three years after hospital discharge. Eight healthy volunteers, matched for age, served as a control group when considering clinical characteristics and investigations on PBMC analysis.

The study was conducted in accordance with the Declaration of Helsinki and approved by the Ethics Committee of the Faculties of Medicine, Dentistry, Pharmacy, Nursing Schools, Physiotherapy, Midwifery and Hospitals of Strasbourg (CE-2020-34, 27 March 2020).

### 4.2. Peripheral Blood Mononuclear Cell (PBMC) Mitochondrial Isolation and Respiration

#### 4.2.1. Peripheral Blood Mononuclear Cell (PBMC) Isolation

Venous blood was sampled in EDTA tubes and deposited on a ficoll density gradient (Eurobio, Lymphocytes separation medium, Courtabeauf France, France) and centrifuged (2100 rpm, 25 min, 18 °C, without brakes). Peripheral blood mononuclear cells were recovered and washed in a Phosphate-Buffered Saline (Hyclone, South Logan, UT, USA) and centrifuged (1600 rpm, 10 min, 18 °C, without brakes). Finally, PBMC were counted by flow cytometry (Muse Cell Analyser, Merck Millipore, Darmstadt, Germany).

#### 4.2.2. Mitochondrial Respiratory Chain Complex Respiration Assessment

**Figure 6:**
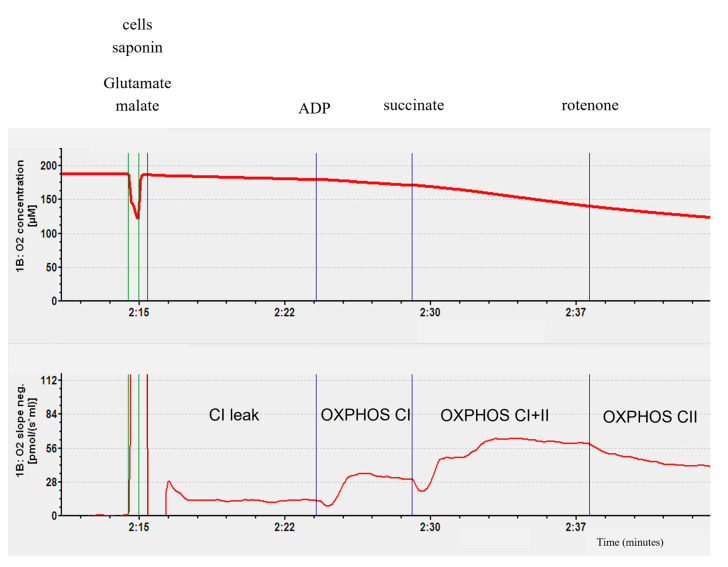
Representative graph obtained during an experiment with the Oroboros system.

To study the mitochondrial respiration, a high-resolution oxygraph (Oxygraph-2k; Oroboros Instruments, Innsbruck, Austria) was used. Briefly, 2.5 × 10^6^ PBMC/mL was introduced in the Oxygraph-2k’s chamber with continuous stirring, at 37 °C. Saponine (50 µg/10^6^ cell) was added to permeabilize the cell membranes, and at the same time, glutamate (5 mM) and malate (2 mM) as substrates for complex I (CI leak). The second step was the ADP injection (2 mM) to stimulate ATP synthase and the oxidative phosphorylation by the complex I (OXPHOX state by CI). The injection of succinate (25 mM) enabled the activation of complex II and obtained an oxidative phosphorylation linked to complexes I and II (OXPHOS state by CI + II). Complex I was then blocked by rotenone (0.5 μM) injection and the oxidative phosphorylation was also linked to complex II (OXPHOS state by CII). The O2 consumption was analyzed using the DatLab software 4.3 (Oxygraph-2k; Oroboros Instruments, Innsbruck, Austria). The result was expressed in pmol/s/10^6^ cell (Figure 6).

The first step is the O2 consumption linked to the injection of cells in the presence of saponin (50 µg/10^6^ cell), glutamate (5 mM), and malate (2 mM). This step reflects O2 consumption passing through complex I without the activation of ATP synthase. It is therefore a non-phosphorylating step, CI leak. The second step is marked by the injection of ADP (2 mM), a substrate of ATP synthase. This step activates oxidative phosphorylation via complex I and is called OXPHOS CI. To activate complex II, succinate is injected into the chamber. This step represents phosphorylating activity passing through complexes I and II, called OXPHOS CI + II. Finally, to obtain phosphorylating activity passing only through complex II, rotenone (0.5 µM), an inhibitor of complex I, is injected. This step is called OXPHOS CII.

### 4.3. RT-PCR

Total RNA was isolated from 5 × 10^6^ PBMC using RNeasy mini kit (Qiagen, Hilden, Germany) according to the manufacturer’s instructions. RNA was quantified by spectrophotometry (Nanodrop, Thermo Fisher). cDNA was synthesized with Maxima™H Minus cDNA synthesis Master Mix (Thermo Fisher Scientific, Waltham, MA, USA), according to the manufacturer’s protocol. Quantitative PCR was performed with 5 ng cDNA, using the QuantStudio 3 Real-Time PCR System (Applied Biosystem, Thermo Fisher Scientific, Waltham, MA, USA) and PowerTrack SYBR Green Master Mix (Applied Biosystem). YWHAZ was used as a housekeeping gene. The expression of genes implicated mainly in inflammatory pathways were analyzed: CXCL9, ISG15, and SUCNR1.

Primer sequences of the genes analyzed in RT-PCR are shown in Table 4 below.

### 4.4. Statistical Analysis

Values are expressed as mean ± standard error of the mean (SEM). Statistical analysis was performed using Prism 6.0 (Graph Pad Software Inc., San Diego, CA, USA). Values were not distributed normally (using the Shapiro–Wilk test), so we performed a non-parametric test (Mann–Whitney test and Kruskal–Wallis test depending on the number of groups to compare). Correlations were analyzed using a non-parametric Spearman’s test. A *p* value < 0.05 was considered significant.

## 5. Conclusions

In conclusion, this study shows that the greater the impairment in PBMC mitochondrial respiration, the greater the mortality rate in patients hospitalized in the intensive care unit for COVID-19. Similarly, impaired PBMC mitochondrial respiration is associated with more severe long-COVID symptoms, three years after hospital discharge. Further, PBMC markers of inflammation also increased with the severity of COVID, and ISG15 negatively correlated with PBMC mitochondrial respiration.

These results support that PBMC mitochondrial respiration might be a biomarker of long-COVID severity, This finding therefore warrants further investigation into whether modulation of PBMC mitochondrial respiration could improve the prognosis of COVID-19 patients.

## Figures and Tables

**Table 1:** Clinical characteristics of the population.

	Healthy Controls	COVID Patients				*p* Value
		Total COVID Patients	With Few Post-COVID Symptoms	With Many Post-COVID Symptoms	Deceased Patients	
Gender (M/F)	5/3	14/6	5/1	3/3	3/1	
Age (years)	59.49 ± 1.10	65.69 ± 1.69	67.22 ± 3.50	63.65 ± 2.30	68.75 ± 4.73	0.055
Body Mass Index (kg/m^2^)	25.0 ± 1.60	29.50 ± 1.33	27.17 ± 1.08	31.33 ± 2.39	29.5 ± 3.62	0.029
	Comorbidities (n, %)					
Hypertension	0	9, 45%	3, 50%	3, 50%	2, 50%	
Dyslipidaemia	0	8, 40%	3, 50%	1, 16.6%	3, 75%	
Diabetes	0	7, 35%	1, 16.6%	5, 83.3%	1, 25%	
Chronic cardiac insufficiency	0	6, 30%	2, 33.3%	3, 50%	1, 25%	
Chronic respiratory insufficiency	0	4, 20%	0, 0%	3, 50%	0, 0%	
Chronic renal insufficiency	0	2, 10%	1, 16.6%	1, 16.6%	0, 0%	

Results are presented as mean ± standard error.

**Table 2:** Biological characteristics of the population.

	Normal Values (Range)	Total COVID Patients	Patients with Few Post-COVID Symptoms	Patients with Many Post-COVID Symptoms	Deceased Patients
Creatinine (umol/L) ± SEM	64–104	111.5 ± 16.40 n = 20	123.5 ± 25.7 n = 6	81.98 ± 31.14 n = 6	129.9 ± 20.64 n = 4
HB (hemoglobin level, g/dL)	12–16	8.93 ± 0.20 n = 20 *p* < 0.0001	9.02 ± 0.35 n = 6 *p* < 0.001	8.67 ± 0.27 n = 6 *p* < 0.0001	8.92 ± 0.64 n = 4 *p* < 0.05
Blood Cell Count (×10^9^/L)					
Leucocytes	4–11	13.52 ± 1.35 n = 20 *p* = 0.08	13.26 ± 1.09 n = 6 *p* = 0.09	11.77 ± 2.46 n = 6	18.33 ± 5.13 n = 4
Lymphocytes	1–4	1.38 ± 0.19 n = 17	1.42 ± 0.12 n = 6	1.82 ± 0.62 n = 5	0.93 ± 0.24 n = 3
Neutrophils	1.40–7.70	8.67 ± 0.82 n = 17	9.13 ± 1.05 n = 6	8.60 ± 1.97 n = 5	9.17 ± 2.25 n = 3
Inflammation					
CRP (mg/L) ± SEM	<5.0	66.38 ± 13.33 n = 12 *p* < 0.001	108.2 ± 33.72 n = 3 *p* = 0.09	60.15 ± 17.33 n = 4 *p* < 0.05	73.43 ± 3.73 n = 3 *p* < 0.01
NLR	1.44–1.63	7.32 ± 1.11 n = 15 *p* < 0.001	6.66 ± 0.89 n = 6 *p* < 0.01	6.39 ± 1.46 n = 5 *p* < 0.05	13.96 6.54 n = 2
LLR	2.35–3.81	0.11 ± 0.01 n = 17 *p* < 0.0001	0.11 ± 0.01 n = 6 *p* < 0.0001	0.12 ± 0.03 n = 6 *p* < 0.0001	0.07 ± 0.01 n = 20 *p* < 0.0001

CRP: C-reactive protein; NLR: neutrophil-to-lymphocyte ratio; LLR: leukocyte-to-lymphocyte ratio. Results are expressed as mean ± standard error of the mean (SEM). COVID patients’ data are compared to the nearest limit of the normal values.

**Table 3:** Patients’ symptoms score three years after hospital release.

Symptoms Score Mean ± SEM/n	Patients with No or Few COVID Symptoms	Patients with Many COVID Symptoms
Anosmia/Agueusia	1/1	6.67 ± 0.88/3
Dyspnea (effort/at rest)	4/1	6.5 ± 0.87/4
Tiredness	6 ± 0/2	5.67 ± 0.8/6
Muscle pain	0/6	5.67 ± 1.02/6
Neural trouble	1.50 ± 0.5/2	4.25 ± 0.75/4
Anxiety	0/6	4.67 ± 0.67/3
Thoracic pain	0/6	9/1
Digestive trouble	0/6	7/1

Results are expressed as mean ± SEM. n is the number of patients presenting with symptoms.

**Table 4:** Sequences of gene-specific primers used for quantitative polymerase chain reaction.

Gene Name	Primer Forward	Primer Reverse
*YWHAZ*	5′-AGGAGATTACTACCGTTACTTGGC-3′	5′-AGCTTCTTGGTATGCTTGTTGTG-3′
*CXCL9*	5′-TAAGCGCTAGAGGAAGCAGC-3′	5′-TTCACTGAACCTCCCCTGGA-3′
*ISG15*	5′-GATCACCCAGAAGATCGGCG-3′	5′-GGATGCTCAGAGGTTCGTCG-3′
*SUCNR1*	5′-CGTTGTGGGAGTCCTTGGAA-3′	5′-GGTTGCTTATGCAGAGCACG-3′

## Data Availability

The original contributions presented in this study are included in the article. Further inquiries can be directed to the corresponding author.
